# Musculoskeletal Health Complaints and Associated Risk Factors in Freshmen Music Students

**DOI:** 10.3390/ijerph20043169

**Published:** 2023-02-10

**Authors:** Nikolaus Ballenberger, Florian Avermann, Christoff Zalpour

**Affiliations:** Faculty of Business Management and Social Sciences, Osnabrück University of Applied Sciences, 49090 Osnabrück, Germany

**Keywords:** music student, risk factor, musculoskeletal health complaints, incidence, prediction, longitudinal

## Abstract

Background: Evidence concerning the development of musculoskeletal health complaints (MHCs) among music students is limited due to inappropriate study designs. We aimed to assess the occurrences of MHCs and associated risk factors in freshmen music students compared to students from other disciplines. Methods: A prospective cohort study was conducted. Risk factors such as pain-related, physical, and psychosocial variables were measured at baseline. Episodes of MHCs were recorded monthly. Results: A total of 146 music students and 191 students from other disciplines were analyzed. In the cross-sectional comparison, pain-related, physical, and psychosocial variables were significantly altered in music students compared to students from other disciplines. Furthermore, music students with current MHCs differed significantly from music students without current MHCs with respect to physical health, pain, and history of MHCs. Our longitudinal analysis showed that monthly MHCs were higher in music students compared to students from other disciplines. Independent predictors for monthly MHCs in music students were current MHCs and reduced physical function. Predictors for MHCs in students from other disciplines were a history of MHCs and stress. Conclusions: We provided insight into the development of MHCs and risk factors in music students. This may help in the creation of targeted, evidence-based prevention and rehabilitation.

## 1. Introduction

Playing-related health complaints may impact practice, rehearsals, performances, and even the musical careers of music students; these complaints are a consequence of a complex interaction of physical, psychological, and psychosocial factors [1,2,3,4]. Among physical playing-related health complaints, musculoskeletal problems are of major importance [5,6]. These are defined as the perception of any discomfort or impairment within the musculoskeletal system, such as pain/problems, weakness, lack of control, numbness, tingling, or other symptoms related to playing [7]. Systematic reviews of studies have summarized the occurrence of musculoskeletal complaints in professional musicians in general and string players specifically. Point prevalence ranges between 9% and 68%, 12-month prevalence between 41% and 93%, and lifetime prevalence between 62% and 93% [5,8]. The high heterogeneity of these occurrences may be explained by different definitions of prevalence and health complaints. Psychological and psychosocial complaints have also been identified, including stress, performance anxiety, and depression (among others) [6,8,9,10,11]. Even at the beginning of their university studies, 79% of music students reported experiences of playing-related pain, and 29% of music students complained about playing-related health issues (both physical and psychological) during their first academic year [12,13]. Various factors have been discussed in the literature as potential predictors of physical health complaints, such as gender, age, professional status, instrument type, number of years playing, hypermobility, stress, and musculoskeletal dysfunction [12,14,15,16,17]. Playing-related musculoskeletal problems are thought to be connected to activity, stress, and pain, as well as psychosocial variables such as performance anxiety [1,18,19,20]. To provide musicians with effective therapeutic interventions and prevention strategies, it is crucial to identify the etiological mechanisms of playing-related complaints. This also requires an understanding of the complex interaction of physical, psychological, and psychosocial factors and the role they play in the development of playing-related complaints.

However, there is only little knowledge of these issues due to aspects such as lack of prospective longitudinal study designs or low study quality [5,16,21]. Most study designs have been cross-sectional, such as surveys measuring a single point in time [16,21]. These study designs present major drawbacks: instead of incidence these only record prevalence. Furthermore, they cannot lead to inferences on the causal relationship between exposure and endpoint (disease) due to the absence of a temporal relationship (exposure is followed by endpoint), which makes it is impossible to identify risk factors. Hence, to measure occurrences of health problems and associated risk factors, longitudinal study designs are necessary.

As a consequence, we launched a prospective cohort study in 2015 at Osnabrück University of Applied Sciences to investigate musculoskeletal health complaints (MHCs) and associated risk factors in freshmen music students. More specifically, we aimed (1) to compare MHCs and MHC-related factors in music students to students from other disciplines at the start of their academic career (cross-sectional analysis); (2) to report occurrences of MHCs within the first academic year; and (3) to identify risk factors and provide a prediction model for the occurrence of MHCs in music students using a prospective longitudinal analysis.

## 2. Materials and Methods

Freshmen music students and freshmen from the same university but from different disciplines were eligible to participate in this prospective cohort study if they met the following criteria: (1) were a first-semester student at Osnabrück University of Applied Sciences; (2) agreed to participate; (3) were at least 18 years old; and (4) were able to communicate in German or English. Both freshmen music students and students from other disciplines were contacted through leaflets and visits to first-semester courses and seminars. Students from other disciplines were recruited from the Faculty of Business Management and Social Sciences. Participants were excluded from the study if the following criteria were relevant: (1) neurological, orthopedic, or psychiatric illness; (2) infectious or systemic diseases; and/or (3) the habitual use of analgesics or psychopharmaceuticals. The study was approved by Osnabrück University of Applied Sciences’ Ethics Review Board, and the article followed the STROBE guidelines [22]. Furthermore, the study followed a pre-planned study process The pilot study by Ballenberger served as a template concerning study design, measurements, data analysis, and definition of MHCs [23]. Furthermore, the data from the pilot study were included in this study. At the start of the study, participants had to fill out a consent letter and a questionnaire regarding their basic demographics and history of health-related problems. Then, two physical therapists administered a battery of tests to assess individuals’ physical and psychological health. These physical therapists were given 10 h of specific training ahead of time by an academic physical therapist with >10 years of clinical experience to familiarize them with all of the testing techniques and increase study reliability. Music students and students from other disciplines received monthly online surveys inquiring about episodes of MHCs, as well as the degree of impairment in daily life (all participants) and when playing their musical instrument (music students only). To improve response rates, reminders were sent out after one week.

### 2.1. Sample

For sample-size calculation, we aimed to identify a one-year incidence rate of 45% with a confidence band ranging from 40% to 50%. Therefore, we calculated a total sample size of 370 [24].

### 2.2. Outcome Measures

#### MHCs

Berque’s modification [19] of Zaza’s original definition [7] for playing-related musculoskeletal disorders was used to measure the occurrence and history of MHCs in music students. The signaling question was: “Do you have the perception of any discomfort or impairment within the musculoskeletal system, such as pain/problems, weakness, lack of control, numbness, tingling, or other symptoms that are related to playing”. An additional question was added: “Do you have the perception of any discomfort or impairment within the musculoskeletal system, such as pain/problems, weakness, lack of control, numbness, tingling, or other symptoms that are related not only to playing but also to daily life”. This provided a variable not limited to musicians alone since we attempted to compare MHCs between musicians and students from other disciplines. The degree to which playing/daily life was affected by MHCs was rated on a 5-point Likert scale from “not at all” (1) to “very severe” (5). For the analysis, we considered MHCs to be present if it was rated at least as “moderate.” For MHCs at baseline (current MHCs), MHCs had to be perceived within the past seven days. A “monthly” prevalence of MHCs was defined as the experience of any MHCs in the previous month that affected daily life at least moderately, and one-year incidences of MHCs were defined as the experience of at least one episode of MHCs that affected daily life at least moderately within the 12 months since starting university. We considered an incident case to be someone who experienced at least one new monthly episode of moderate MHCs within the past year. A case was counted as “new” if subjects had no current MHC at baseline but then reported an episode of MHCs afterward or if a subject reported at least one month without MHCs between two episodes of MHCs.

Demographic data: we developed a questionnaire with the following characteristics: age, height, weight, BMI, weekly sports in hours (h), hours of sleep, tobacco use, alcohol consumption, and sex. In addition, music students were asked several questions about their practice habits (e.g., playing time in h, main musical instrument).

Core strength endurance: core strength endurance was measured by conducting front planks and the extensor endurance Biering-Sørensen (EEBS) test [25,26]. In both tests, subjects held the position until fatigue or any other reason caused them to terminate the test, and the time was recorded. The core strength endurance tests are considered intrareliable (ICC: 0.95–0.97) and valid (Pearson’s correlation: 0.52–0.97) [27,28,29].

Hypermobility: general hypermobility was assessed using the Beighton score, ranging from 0 to 9 points, indicating very low to very high general hypermobility [30]. This test demonstrates inter- and intrareliability (ICC: 0.49–0.94; Spearman’s rho: 0.86 and 0.87) [31,32]. The Beighton score is valid compared to measurement with a goniometer (*p*-values of ANOVA: <0.001–0.06) [33].

Cervical range of motion (CROM): the active range of joint motion of the cervical spine was more generally assessed using a cervical range-of-motion goniometer (CROM, Baseline Evaluation Instruments by Fabrication Enterprises Inc. of White Plains, NY, USA). The CROM device is considered valid (Pearson’s correlation: 0.93–0.98) [34], inter-reliable (ICC: 0.56–0.92), and intrareliable (ICC: 0.75–0.98) [34,35].

Mechanosensitivity: subjects’ mechanical pressure pain threshold (PPT) was assessed over predefined anatomical points for various muscles of the upper and lower extremities, the trunk, and the head, including musculus gastrocnemius lateralis, m. semi spinalis capitis, m. levator scapulae, m. trapezius pars transversus, the origin of wrist extensors, the origin of wrist flexors, m. sternocleidomastoideus, m. masseter, and m. temporalis. Measurements were performed using an algometer (model FPK5, Wagner Instruments, Greenwich, CT, USA). The pressure was increased at a constant rate of approximately 1 kg/cm^2^/s. The value at which the sensation of “pressure” changed to one of “pain or discomfort” was recorded. Three measurements were taken for each muscle and averaged for analysis. PPT is a valid (Pearson’s correlation: 0.99) and intrareliable (ICC: 0.7–0.94) method used to measure mechanosensitivity [36,37].

Health-related quality of life: the Short-Form Health Survey 12 (SF12) was used to measure health-related quality of life. Eight sub-scores addressed physical and mental health [38,39,40]. The assessment is considered valid (Pearson’s correlation: 0.38–0.56) and test-retest-reliable (ICC: 0.6–0.78) [41].

Stress and stress symptoms: the Stress and Coping Inventory (SCI) was used to evaluate stress and stress symptoms. For our purposes, only the dimension of stress symptoms was analyzed. We also used the Hospital Anxiety and Depression Scale (HADS). This questionnaire is valid (correlation coefficient: 0.67–0.77) and reliable (Cronbach’s alpha: 0.68–0.93) [42].

Perfectionism: finally, we used the Frost Multidimensional Perfectionism Scale—German (FMPS-D) to evaluate subjects’ perfectionism. The FMPS-D is a valid (correlation coefficient: 0.57–0.85) and reliable (Cronbach’s alpha: 0.77–0.93) instrument [43].

Performance anxiety: for the group of freshman music students, we conducted the Kenny Music Performance Anxiety Inventory (K-MPAI) test to assess performance anxiety. It is considered to be valid (correlation coefficient: 0.12–0.88) and intrareliable (Cronbach’s alpha: 0.92) [11,44,45,46].

### 2.3. Statistical Analysis

The statistical analysis adhered to the analysis strategy described in the pilot study by Ballenberger et al. [23]. All statistical analyses were performed using R (ver. 3.6.3, R Development Core Team, Vienna, Austria, 2020). A *p*-value of <0.05 was considered significant if not stated otherwise. We compared the study group’s baseline characteristics and demographic data using Chi-square tests and independent *t*-tests for categorical and continuous data, respectively. We also determined descriptive statistics such as means, standard deviations, and counts (percentages).

For the baseline analysis, we used univariate linear regression and logistic regression models. The effects are given as standardized mean differences (SMD) and odds ratios (OR), which made it possible to compare effects across different outcome measurements.

The goal of the univariate longitudinal analysis was to identify risk factors (predictors) for the emergence of monthly MHCs in music students and compare them to those in students from other disciplines. Here, we employed a general linear mixed model with binomial distribution to account for the correlated data structure (repeated measurements). The effects are given as odds ratios. In addition, we modeled the interaction between groups (music students and students from other disciplines) and the history of MHCs and health-related factors to evaluate whether risk factors for monthly MHCs differed significantly between groups. Here, a *p*-value of <0.1 was considered significant in order to gain power and not miss true interaction effects [47]. All univariate regression models were adjusted for the covariates of age and gender since these were considered potential confounders.

The goal of the multivariate longitudinal analysis was to identify a set of predictors/risk factors for monthly events of MHCs in music students. Based on the univariate longitudinal analysis, we entered potential candidate variables for the prediction model if their *p*-value was <0.1. The predictive accuracy of the model was determined by calculating the area under the ROC curve (AUCs).

We employed residual diagnostics and plots to check the validity of all regression models.

## 3. Results

From 2015 to 2020, six cohorts totaling 337 subjects (146 music students (69 female) and 191 students (100 female) from other disciplines) participated in the study. The mean age of the music students and students from other disciplines was 21.6 ± 3.17 and 22.88 ± 3.82 (*p* < 0.05), respectively. Students from other disciplines were mainly freshmen from health sciences, business sciences, and social work, representing a cross-section of the faculty. Measurements were taken during the first three months of each cohort’s first semester. The first monthly online questionnaire was sent out directly after the completion of baseline measurements. Out of 337 subjects, 190 (97 music students and 93 students from other disciplines) completed at least one monthly questionnaire. This corresponds to a total response rate of 56% (66% in music students and 49% in students from other disciplines). A total of 1231 out of 2280 (190 × 12 months) maximally possible monthly completed questionnaires were completed. This corresponds to a completion rate of 54% in both cohorts. On average, music students completed 6.20 ± 4.08 monthly questionnaires, and students from other disciplines completed 6.20 ± 4.52 monthly questionnaires, respectively. In each cohort, 10% of the participants completed the monthly questionnaires fully (12 months).

### 3.1. Cross-Sectional Analysis: Comparison between Music Students and Students from Other Disciplines

Table 1 shows differences in all measured variables between music students and students from other disciplines at baseline (cross-sectional analysis). It also presents means and SDs for continuous variables, as well as absolute numbers and percentages for categorical variables. Finally, the table depicts adjusted differences between these two groups, represented by standardized mean differences (SMDs) and odds ratios (ORs), respectively, depending on the data type (continuous vs. categorical).

Compared to students from other disciplines, music students had more body weight and were less active in sports. They reported less physical functioning (SF12), higher pain (SF12), and, more often, current MHCs. Furthermore, mental health (SF12) was lower, while stress symptoms, anxiety, and depression scores were higher. In addition, music students were more perfectionistic. All differences were significant. Effect sizes ranged from small to moderate. Other comparisons, such as hypermobility, core strength endurance, cervical range of motion, and mechanosensitivity, were insignificant (Table S1).

### 3.2. Comparison between Music Students with and without Current MHCs

Differences between music students with and without current MHCs (presence of MHCs during the last seven days) at baseline (cross-sectional comparison) are given in Table 2.

When comparing music students with current MHCs to music students without current MHCs, pain and physical functioning (both SF12) were significantly increased. In addition, a history of MHCs was more frequent among music students with current MHCs. Music students with current MHCs tended to be more anxious and depressed. However, these findings were not significant. The variables of perfectionism and performance anxiety were not significantly different. Other comparisons, such as hypermobility, core strength endurance, cervical range of motion, and mechanosensitivity, were also insignificant (Table S2).

### 3.3. Longitudinal Analysis

#### Monthly Episodes of MHCs

We recorded 1231 monthly episodes of MHCs (monthly prevalence) among students in their first academic year (Table 3). This corresponds to an overall predicted probability for monthly MHCs of 0.13. When divided into subgroups, the predicted probability for music students amounted to 0.17 (149 out of 627 records were positive, and 455 were negative). In other words, the probability of a music student reporting an episode of MHCs in the upcoming month was 17%. The predicted probability for students from other disciplines was 0.09 (107 out of 604 records were positive and 520 were negative).

The number of subjects and the predicted probability of at least one monthly episode of MHCs within the first academic year (incidence) are given in Table S3 in the Supplementary Material.

### 3.4. Risk Factors and Prediction Model

Table 4 depicts the results of the univariate longitudinal analysis of all risk factors (predictors) of developing MHCs over time. The results for each predictor are presented separately for music students and students from other disciplines. The *p*-values of the interaction indicate whether the results of the respective predictor are significantly different between both groups.

In students from other disciplines, significant predictors from baseline included reduced mental health, higher mechanosensitivity, and the number of stress symptoms. Increased anxiety and a history of MHCs were also associated with a higher chance of experiencing MHCs over time, although this increase was not significant. In music students, the results were different. Significant predictors from baseline were increased bodily pain, reduced physical functioning, current MHCs, and previous MHCs (four weeks). Furthermore, MHCs ever, MHCs in the previous year, and increased perfectionism were associated with a higher chance of experiencing MHCs over time. In contrast, longer lifetime play was associated with less chance of developing MHCs. However, these findings did not reach significance. Other variables, such as hypermobility, core strength endurance, cervical range of motion, and mechanosensitivity, were also not significant predictors (Supplementary Materials).

As the significant *p*-values of the interaction terms indicate, the results of the predictors of mental health, physical functioning, and the number of stress symptoms were significantly different between groups; this suggests a different risk profile in music students compared to students from other disciplines.

The results of the multivariate longitudinal analysis (prediction model) are presented in Table 5. This table considers all significant predictors from the univariate analysis (Table 4). The final set of significant independent predictors for MHCs in music students were current MHCs and reduced physical function. In contrast, the final set of significant independent predictors for MHCs in students from other disciplines were a history of MHCs in the previous year and increased stress symptoms.

**Figure 1:**
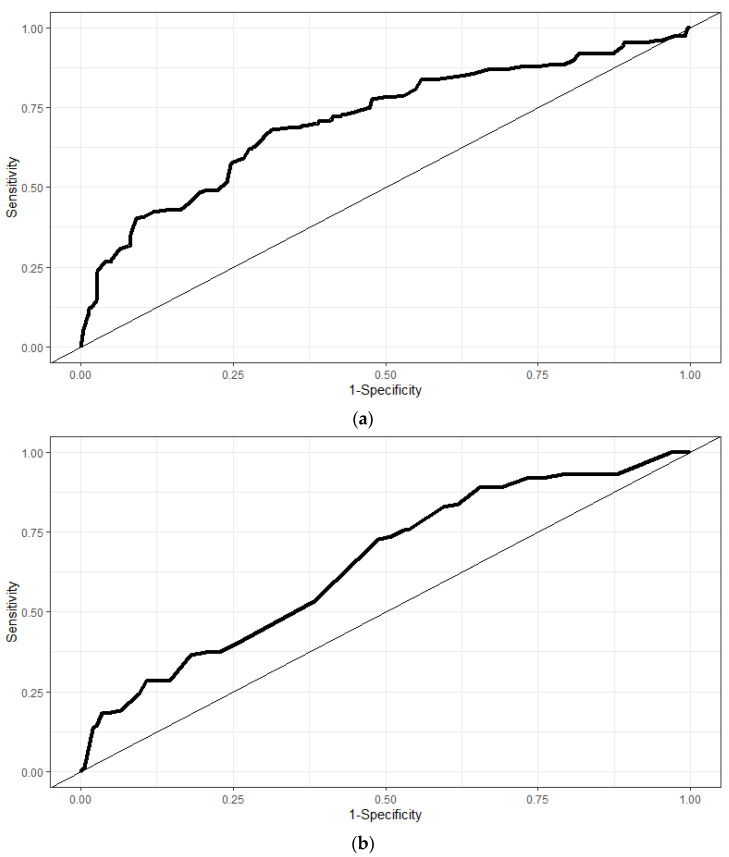
The predictive ability of prediction models investigating MHC in music students (**a**) and in students from other disciplines (**b**).

The predictive ability of both models is depicted in Figure 1a,b. The corresponding AUC for MHCs in music students amounts to 0.72 (*p* < 0.001) and 0.67 (*p* < 0.001) in students from other disciplines, respectively. The results confirm the presence of different risk profiles between music students and students from other disciplines.

## 4. Discussion

In this work, we presented the results from a prospective longitudinal study with the overall goal of assessing MHCs and related risk factors in freshmen music students within their first academic year. We analyzed data from six cohorts totaling 146 music students and 191 students from other disciplines. Our cross-sectional analysis revealed that, at baseline, music students were less active in sports compared to students from other disciplines. Physical functioning (SF12) was reduced; pain (SF12) and history of MHCs were higher. Furthermore, mental health (SF12) was decreased, and stress symptoms, anxiety, and depression scores were increased. These findings are in line with other research. Results from a Canadian study sample showed that music students have poorer mental and physical health than non-music controls [48]. Furthermore, Steinmetz et al. reported more painful body regions and musculoskeletal dysfunctions in music students compared to non-musicians [15]. Similar results were found by Kok et al., where music students also reported more musculoskeletal complaints [49]. Spahn found that both anxiety and depression were significantly higher in music students compared to sports students [50].

Furthermore, our cross-sectional analysis revealed that music students with current MHCs suffered from pain (SF12) significantly more, their physical function was diminished, and they were more likely to report a history of MHCs compared to music students without current MHCs. In addition, music students with current MHCs tended to be more anxious and depressed. Similar results based on our longitudinal data corroborated this. In music students, single significant predictors at baseline for episodes of MHCs within students’ first academic year were increased physical pain, less physical functioning, the presence of MHCs, and a history of MHCs.

Reported risk factors for MHCs from other research studies are only partly in line with our findings. Other studies found previous musculoskeletal injuries or complaints and self-rated health as associated factors with MHCs [15,50], while, as also seen in our data, exercise behavior and cigarette smoking were unrelated to MHCs [16]. However, in contrast to other research, we could not confirm music performance anxiety or high levels of stress as relevant predictors [16]. Instead, we additionally identified increased bodily pain and reduced physical functioning as predictors for MHCs. Nonetheless, a comparison of the risk factors we identified to those from other research is of limited value because our results, in contrast to other studies, are, on the one hand, based partly on prospective longitudinal data and, on the other hand, adjusted for confounders such age and gender—which are often lacking in other studies [15,20].

Interestingly, we found that risk factors for MHCs were different in music students compared to students from other disciplines, leading to the hypothesis that music students and students from other disciplines have distinct risk profiles for MHCs. In students from other disciplines, single significant predictors were poorer mental health, higher mechanosensitivity, and increased stress symptoms. Increased anxiety and a history of MHCs were also positively associated with MHCs over time, but these findings were not statistically significant. Furthermore, a history of MHCs ever, MHCs in the previous year, and increased perfectionism were all associated with a higher chance of experiencing MHCs over time. Significant interaction terms and results from multivariate longitudinal analysis support the assumption of different risk profiles between the two groups. Based on results from the multivariate longitudinal analysis, significant independent predictors for MHCs in music students were current MHCs and reduced physical function, while the final set of significant independent predictors for MHCs in students from other disciplines was a history of MHCs in the previous year and increased stress symptoms. Our results suggest that risk factors for music students are physical-health-oriented, such as bodily pain and physical function, while risk factors for students from other disciplines are more closely related to mental health, such as stress symptoms or anxiety. The presence or history of MHCs is a risk factor common to both of our cohorts. These findings are in line with other research [51,52], where both stress and a prior history of lower back pain were found to be independent predictors for chronic pain or lower back pain in young students.

Additional results from the longitudinal data showed that the probability of MHCs within subjects’ first academic year is substantially higher for music students than students from other disciplines. However, our risk factor analysis results regarding the occurrence of MHCs (monthly records and yearly incidence) cannot be compared to other studies because previous studies were not based on longitudinal data or did not use a similar definition of MHCs [51,53]. We could not find any comparison data in the literature for incidences. Only our data for current pain at baseline serves as a reference value for point prevalence. Here, our estimated value of 27% corresponds to other research [5,20].

### 4.1. Strengths and Weaknesses

A variety of limitations need to be discussed. Thus far, we have investigated MHCs and their corresponding risk factors in a rather general way. We defined MHCs as the perception of any discomfort or impairment within the musculoskeletal system, such as pain/problems, weakness, lack of control, numbness, tingling, or other symptoms. We neither differentiated health complaints within specific parts of the body nor provided a risk factor analysis for specific subgroups of performing artists, such as string instrument or wind players. Here, risk profiles and occurrences of MHCs might be distinct when stratified accordingly, as other research has shown [54]. Furthermore, subgrouping could have led to more in-depth results, which would have potentially helped disentangle the complex interactions of physical, psychological, and psychosocial factors and their role in the development of MHCs. Hence, omitting subgrouping might have resulted in ignoring/overlooking the underlying interaction. However, more participants would be required for valid subgroup analysis. Our results might also be explained by selection bias and/or non-random missing data. The study population might not be representative of the source population. For instance, the group of non-music students might be overrepresented by more healthy subjects than the general non-musician source population, or the music students might be overrepresented by less-healthy subjects compared to the general source population of music students. Similarly, music students with MHCs might have completed the monthly questionnaires systematically differently than those without MHCs. Hence, an overestimation or underestimation, respectively, of occurrences of MHCs might be the result. Another limitation of our study is related to our definition of MHCs. Even though we adhered to Berque’s modification [19] of Zaza’s original definition, we had to slightly expand it to encompass not only playing but also daily life. This provided us with a variable that would not be limited to musicians alone, as we sought to compare MHCs between musicians and students from other disciplines. However, this also led to the drawback of limited comparability to other research and might contribute to the large heterogeneity of study results regarding the occurrences of MHCs in musicians and the performing arts.

Finally, our results may only be valid for freshmen or music students in general. Generalization to other populations of professional musicians, such as orchestra musicians, is unclear, as these were not included in our analysis.

The strength of this study lies in the unique nature of our longitudinal study design. This allowed us to prospectively track monthly episodes of MHCs and properly investigate the risk factors. To our knowledge, this study is the first in the field of musicians’ health. Another study with a similar purpose is being conducted at the moment [55]. Furthermore, the large sample size allowed us to apply detailed data analysis. By using multivariate regression analysis, we were able to detect independent risk factors by adjusting for covariates. Furthermore, we succeeded in building a prediction model and modeling interaction terms to detect varying risk profiles between music students and students from other disciplines.

### 4.2. Clinical Implications and Research Directions for the Future

The knowledge of relevant and modifiable risk factors for MHCs, such as current pain and reduced physical function at the beginning of the academic music career, has clear clinical implications for the health of music students. For instance, music students might be screened with respect to underlying risk factors at various stages of their academic career such as at the beginning of their studies and after certain intervals. This helps to identify music students at risk for MHCs at an early stage. Furthermore, knowledge of risk factors forms the basis for the content of targeted, evidence-based prevention programs and rehabilitation. As indicated by our results, such programs should strongly focus on increasing physical function and reducing pain, e.g., providing music students, especially those at risk, with targeted, evidence-based prevention programs alongside their academic career, which might reduce their individual burden of disease, improve their health-related quality of life, and hence optimize practicing time and rehearsals with better health. Thus far, there is little evidence about the effectiveness of specific therapeutic strategies to treat or prevent MHCs. As far as we know, only one single RCT has attempted to assess the effect of a biopsychosocial prevention course tailored to musicians, finding that it was not superior to physical activity promotion in reducing disability from MHCs in music students [56]. This finding might be explained, at least in part, by the lack of knowledge about proper risk factors based on longitudinal study designs. Accordingly, as seen in our study, the role of psychosocial factors for the development of MHC is unclear. Variables such as anxiety and depression were only weakly associated with MHC at the cross-sectional analysis but not with the development of MHC at the longitudinal analysis. As a consequence, addressing those in prevention programs might prove ineffective.

Hence, for the development of effective prevention programs and rehabilitation, more in depth research is urgently needed. In the future, more prospective longitudinal studies need to be conducted in this field of research to replicate and validate our results. Furthermore, these studies should focus on MHCs of specific parts of the body, such as spinal pain or shoulder complaints, and provide a risk factor analysis for specific subgroups of performing artists. This will help to optimize targeted evidence-based prevention programs and rehabilitation tailored to address specific MHC depending on the type of instrument. Here, risk profiles and consequently prevention strategies are expected to be distinct, e.g., violin players with risk for shoulder pain will require different prevention strategies compared to drum players with risk for low back pain.

## 5. Conclusions

The results of our prospective cohort study provided new insights into the occurrence and development of MHCs and the corresponding risk factors in freshmen music students. Freshmen music students were at higher risk of developing episodes of MHCs within their first academic year than students from other disciplines. Furthermore, we identified a set of risk factors and a prediction model that implies a distinct risk profile for music students compared to students from other disciplines. Our study results provide the foundation for developing effective targeted and evidence-based prevention programs and rehabilitation, which is crucial for music students, who represent a population that suffers disproportionately from MHCs.

## Figures and Tables

**Table 1:** Differences between music students and students from other disciplines at baseline (cross-sectional analysis at baseline) represented by standardized mean differences (SMDs) and odds ratios (ORs), respectively, depending on data type (continuous vs. categorical).

Continuous Variables	Music Students			Students from Other Disciplines			SMD *	*p*-Value *
	N	Mean	SD	N	Mean	SD		
**Weight** (kg)	145	70.17	11.96	189	67.35	11.66	**−0.34**	**<0.01**
**Height** (cm)	145	173.26	22.45	190	172.25	16	−0.07	0.56
**BMI**	145	22.65	5.53	190	22.35	2.21	−0.21	0.06
**Sports** (h/week)	142	3.25	3.88	189	5.28	6.09	**0.35**	**<0.01**
**Practice duration** (h/week)	137	19.78	13	NA	NA	NA	NA	NA
**Health-related quality of life** (SF12)								
Physical functioning	139	52.58	6.64	178	54.96	5.05	**0.41**	**<0.01**
Mental health	139	46.98	9.87	178	49.95	8.18	**0.31**	**0.01**
Pain	147	1.66	0.74	189	1.3	0.56	−**0.5**	**<0.01**
**Anxiety and Depression scale** (HADS)								
Depression	98	3.39	2.84	122	2.3	2.69	−**0.42**	**<0.01**
Anxiety	98	6.11	3.59	122	4.54	2.84	−**0.48**	**<0.01**
**Perfectionism** (FMPS)	68	48.19	32.89	63	41.17	31.28	−**0.35**	**0.05**
**Stress symptoms** (SCI)	130	23.71	5.98	151	20.4	4.85	−**0.65**	**<0.01**
**Performance anxiety** (K-MPAI)	97	91.39	36.34	NA	NA		NA	NA
**PPT**	98	11.98	12.43	111	11.15	11.84	−0.06	0.68
**Categorical Variables**	**N**	**Absolute Numbers**	**%**	**N**	**Absolute Numbers**	**%**	OR *	*p*-Value *
**Pain history** (yes/no)								
Last 7 days	131	36/95	27	148	23/125	16	**2.27**	**0.01**
Last 4 weeks	131	47/84	36	147	43/104	29	1.41	0.22
Last 12 months	131	70/61	53	149	75/74	50	1.12	0.66
Ever	131	92/31	70	149	102/47	68	1.22	0.49

* Adjusted for age and gender. Bold SMD, OR, and *p*-values mean significant findings.

**Table 2:** Differences between music students with MHCs and those without MHCs (cross-sectional analysis at baseline) represented by standardized mean differences (SMDs) and odds ratios (ORs), respectively, depending on data type (continuous vs. categorical).

Continuous Variables	Without Current MHCs			With Current MHCs			SMD *	*p*-Value *
	N	Mean	SD	N	Mean	SD		
**Weight** (kg)	94	71.11	11.93	36	69.92	11.43	−0.18	0.32
**Height** (cm)	94	173.18	27	36	175.83	9.69	0.12	0.56
**BMI**	94	22.61	3.31	36	22.54	3.39	−0.08	0.56
**Sports** (h/week)	91	3.58	4.4	36	3	2.9	−0.15	0.46
**Practice duration** (h/week)	88	21.37	13.01	34	19.49	14.27	−0.11	0.58
**Health-related quality of life** (SF12)								
Physical functioning	**87**	**54.29**	**4.94**	**36**	**47.56**	**7.93**	−**1.00**	**<0.01**
Mental health	87	48.4	8.83	36	45.64	10.4	−0.29	0.12
Pain	**95**	**1.39**	**0.53**	**36**	**2.29**	**0.81**	**1.22**	**<0.01**
**Anxiety and Depression scale** (HADS)								
Depression	70	3.06	2.69	27	4.15	3.11	0.39	0.09
Anxiety	70	5.67	3.5	27	7.11	3.65	0.41	0.07
**Perfectionism** (FMPS)	51	48	34.38	16	51.13	28.09	0.03	0.9
**Stress symptoms** (SCI)	95	23.22	5.53	34	25.03	7.1	0.32	0.11
**Performance anxiety** (K-MPAI)	70	89.59	36.67	26	94.87	35.88	0.15	0.51
**PPT**	68	12.85	13.33	30	9.99	10	−0.22	0.31
**Categorical Variables**	**N**	**Absolute Numbers**	**%**	**N**	**Absolute Numbers**	**%**	OR *	*p*-Value *
**Pain history** (yes/no)								
Last 4 weeks	**95**	**14/81**	**15**	**36**	**33/3**	**92**	**84.36**	**<0.01**
Last 12 months	**95**	**38/57**	**40**	**36**	**32/4**	**89**	**13.01**	**<0.01**
Ever	**95**	**60/35**	**63**	**36**	**32/4**	**89**	**5.25**	**<0.01**

* Adjusted for age and gender. Bold SMD, OR, and *p*-values mean significant findings.

**Table 3:** Number of monthly episodes of MHCs within subjects’ first academic year and predicted probabilities of MHCs.

		MHCs			Predicted Probabilities
		**No**	**Yes**	**All**	
**Music students** **Students from other disciplines**	**Yes**	455	149	627	0.17
	**No**	520	107	604	0.09
	**All**	975	256	1231	0.13

**Table 4:** Univariate longitudinal analysis of risk factors (predictors) for developing MHCs. *p*-values of the interaction terms indicate whether ORs of the predictors are significantly different between groups.

	Monthly Episodes: Students from Other Disciplines			Monthly Episodes: Music Students			Interaction *p*-Value *
	N	OR	*p*-Value *	N	OR	*p*-Value *	
**Weight** (kg)	83	1.02	0.35	87	1	1	0.99
**Height** (cm)	83	1.01	0.8	87	0.99	0.75	0.98
**BMI**	83	1.11	0.44	87	0.98	0.76	0.94
**Sports** (h/week)	83	0.97	0.82	85	1.01	0.84	0.84
**Practice duration** (h/week)	NA	NA	NA	82	1	0.78	NA
**Health-related quality of life** (SF12)							
Physical functioning	83	0.99	0.91	86	**0.89**	**<** **0.01**	**0.02**
Mental health	83	**0.93**	**0.02**	86	0.99	0.71	**0.09**
Pain	82	1.92	0.16	88	**2.68**	**<0.01**	0.2
**Anxiety and Depression scale** (HADS)							
Depression	57	1.09	0.26	60	1.07	0.49	0.57
Anxiety	83	1.17	0.06	60	1.07	0.37	0.29
**Perfectionism** (FMPS)	NA	NA	NA	33	1.02	0.09	NA
**Stress symptoms** (SCI)	57	**1.17**	**<0.01**	87	1.02	0.67	**0.03**
**Performance anxiety** (K-MPAI)	NA	NA	NA	60	0.99	0.34	NA
**PPT**	72	0.95	0.05	78	0.99	0.7	0.39
**Pain history** (yes/no)							
Last 7 days	73	2.76	0.12	88	**5.84**	**<0.01**	0.22
Last 4 weeks	82	1.85	0.28	88	**3.03**	**0.01**	0.26
Last 12 months	82	2.92	0.06	88	1.98	0.09	0.8
Ever	73	2.58	0.15	88	2.08	0.09	0.95

* Adjusted for age and gender. Bold OR, *p*-values and Interaction *p*-Value mean significant findings.

**Table 5:** Multivariate longitudinal analysis of risk factors for developing MHCs (prediction model).

Students from Other Disciplines	OR	*p*-Value	Music Students	OR	*p*-Value
MHCs (last 12 months)	2.43	0.05	Current MHCs	2.8	0.01
Stress symptoms	1.15	0.004	Physical function	0.91	0.001
Model *p*-value		0.002	Model *p*-value		<0.001

## Data Availability

The data presented in this study are available on request from the corresponding author. The data are not publicly available because the research is still ongoing.

## Supplementary Materials

The following supporting information can be downloaded at: https://www.mdpi.com/article/10.3390/ijerph20043169/s1, Table S1: Differences between music students and students from other disciplines at baseline (Cross-sectional analysis at baseline), Table S2: Differences between music students with MHC and those without MHC (Cross-sectional analysis at baseline), Table S3: Number of subjects and the predicted probability of their reporting at least one monthly episode of MHC within their first academic year, Table S4: Univariate longitudinal analysis of risk factors (predictors) for developing MHC.
