# Supplementary material for: Musculoskeletal Health Complaints and Associated Risk Factors in Freshmen Music Students
Source: Int J Environ Res Public Health. 2023 Feb 10;20(4):3169. doi: 10.3390/ijerph20043169 (PMC9964731; doi:10.3390/ijerph20043169)
Supplement: Supplementary file 1 [file ijerph-20-03169-s001.zip › ijerph-2178702-supplementary.pdf]

Table S1: Differences between music students and students from other disciplines at baseline (Cross-sectional analysis at baseline)

Continuous variables	Music students			Students from other disciplines			p-value	SMD*	Confidence interval*		p-value*
	N	Mean	SD	N	Mean	SD			lower	upper	
**Age** (years)	144	21.6	3.17	186	22.88	3.82	**<0.01**	-0.36	-0.58	-0.15	**<0.01**
**Weight** (kg)	145	70.17	11.96	189	67.35	11.66	**0.03**	-0.34	-0.55	-0.13	**<0.01**
**Height** (cm)	145	173.26	22.45	190	172.25	16	0.65	-0.07	-0.29	0.16	0.56
**BMI**	145	22.65	5.53	190	22.35	2.21	0.37	-0.21	-0.43	0.001	0.06
**Sleep duration** (h)	146	7.25	0.93	190	7.26	0.77	0.89	0.06	-0.17	0.28	0.62
**Sports** (h/week)	142	3.25	3.88	189	5.28	6.09	**<0.01**	0.35	0.13	0.56	**<0.01**
**Tobacco use** (cigarettes/day)	146	0.95	2.63	190	0.66	2.55	0.31	-0.17	-039	0.05	0.14
**Practice duration** (h/week)	137	19.78	13	NA	NA	NA	NA	NA	NA	NA	NA
**Experience with primary instrument** (years)	143	11.14	4.73	NA	NA	NA	NA	NA	NA	NA	NA
**Health-related quality of life** (SF12)											
Physical functioning	139	52.58	6.64	178	54.96	5.05	**<0.01**	0.41	0.19	0.63	**<0.01**
Mental health	139	46.98	9.87	178	49.95	8.18	**<0.01**	0.31	0.09	0.54	**0.01**
Pain	147	1.66	0.74	189	1.3	0.56	**<0.01**	-0.5	-0.71	-0.29	**<0.01**
**Anxiety and Depression scale** (HADS)											
Depression	98	3.39	2.84	122	2.3	2.69	**<0.01**	-0.42	-0.7	-0.14	**<0.01**
Anxiety	98	6.11	3.59	122	4.54	2.84	**<0.01**	-0.48	-0.75	-0.2	**<0.01**
**Perfectionism** (FMPS)	68	48.19	32.89	63	41.17	31.28	0.21	-0.35	-0.69	-0.01	**0.05**
**Stress symptoms** (SCI)	130	23.71	5.98	151	20.4	4.85	**<0.01**	-0.65	-0.9	-0.41	**<0.01**

**Performance anxiety** (K-MPAI)	97	91.39	36.34	NA	NA	NA	NA	NA	NA	NA	NA
**Hypermobility** (Beighton score)	113	2.25	2.29	150	2.11	2.07	0.62	-0.07	-0.32	0.18	0.6
**Total cervical range of motion**	98	65.36	6.7	111	66.24	7.16	0.36	0.15	-0.14	0.45	0.31
**Holding time, front plank** (sec)	110	83.29	43.88	149	91.19	54.04	0.2	0.12	-0.12	0.37	0.33
**Holding time, EEBS test** (sec)	113	122.14	54.94	149	132.83	47.73	0.1	0.2	-0.04	0.45	0.11
**PPT**	98	11.98	12.43	111	11.15	11.84	0.62	-0.06	-0.36	0.24	0.68
Categorical variables	**N**	**Absolute numbers**	**%**	**N**	**Absolute numbers**	**%**	**p-value**	**OR***	**Confidence interval***		**p-value***
									**Lower**	**Upper**	
**Sex** (male/female)	146	77/69	53	191	91/100	48	0.3	0.8	0.35	1.25	0.3
**Pain history** (yes/no)											
Last 7 days	131	36/95	27	148	23/125	16	**0.02**	2.27	1.21	4.34	**0.01**
Last 4 weeks	131	47/84	36	147	43/104	29	0.29	1.41	0.82	2.43	0.22
Last 12 months	131	70/61	53	149	75/74	50	0.64	1.12	0.68	1.87	0.66
Ever	131	92/31	70	149	102/47	68	0.7	1.22	0.7	2.12	0.49

* Adjusted for age and sex

Table S2: Differences between music students with MHC and those without MHC (Cross-sectional analysis at baseline)

Continuous variables	Without current MHC			With current MHC			p-value	SMD*	Confidence Interval*		p-value*
	N	Mean	SD	N	Mean	SD			Lower	Upper	
**Age** (years)	93	21.75	3.25	36	22.22	2.83	0.42	0.15	-0.19	0.79	0.56
**Weight** (kg)	94	71.11	11.93	36	69.92	11.43	0.6	-0.18	-0.55	0.18	0.32
**Height** (cm)	94	173.18	27	36	175.83	9.69	0.41	0.12	-0.29	0.53	0.56
**BMI**	94	22.61	3.31	36	22.54	3.39	0.92	-0.08	-0.43	0.27	0.56
**Sleep duration** (h)	95	7.27	0.75	36	7.42	0.87	0.37	0.17	-0.16	0.5	0.31
**Sports** (h/week)	91	3.58	4.4	36	3	2.9	0.39	-0.15	-0.56	0.25	0.46
**Tobacco use** (cigarettes/day)	95	1.09	2.81	36	0.99	2.61	0.85	-0.07	-0.47	0.34	0.76
**Practice duration** (h/week)	88	21.37	13.01	34	19.49	14.27	0.51	-0.11	-0.52	0.29	0.58
**Experience with primary instrument** (years)	96	11.26	4.84	35	11.76	4.77	0.6	0.04	-0.3	0.37	0.84
**Health-related quality of life** (SF12)											
Physical functioning	87	54.29	4.94	36	47.56	7.93	**<0.01**	-1	-1.35	-0.66	**<0.01**
Mental health	87	48.4	8.83	36	45.64	10.4	0.17	-0.29	-0.66	0.08	0.12
Pain	95	1.39	0.53	36	2.29	0.81	**<0.01**	1.22	0.89	1.54	**<0.01**
**Anxiety and Depression scale** (HADS)											
Depression	70	3.06	2.69	27	4.15	3.11	0.12	0.39	-0.05	0.84	0.09
Anxiety	70	5.67	3.5	27	7.11	3.65	0.09	0.41	-0.03	0.86	0.07
**Perfectionism** (FMPS)	51	48	34.38	16	51.13	28.09	0.72	0.03	-0.53	0.59	0.9
**Stress symptoms** (SCI)	95	23.22	5.53	34	25.03	7.1	0.18	0.32	-0.07	0.71	0.11

**Performance anxiety** (K-MPAI)	70	89.59	36.67	26	94.87	35.88	0.53	0.15	-0.3	0.61	0.51
**Hypermobility** (Beighton score)	68	2.4	2.43	30	2.47	2.15	0.89	0.05	-0.4	0.49	0.84
**Total cervical range of motion**	68	65.24	6.71	30	65.63	6.79	0.79	0.05	-0.39	0.48	0.83
**Holding time, front plank** (sec)	66	80.62	41.29	29	103.21	46.81	**0.03**	0.52	0.09	0.95	**0.02**
**Holding time, EEBS test** (sec)	68	126.21	52.25	30	132.7	59.42	0.61	0.14	-0.28	0.57	0.51
**PPT**	68	12.85	13.33	30	9.99	10	0.24	-0.22	-0.65	0.2	0.31
Categorical variables	**N**	**Absolute numbers**	**%**	**N**	**Absolute numbers**	**%**	**p-value**	**OR***	**Confidence interval* Lower**	**Confidence interval* Upper**	**p-value***
**Sex** (male/female)	95	51/44	54	36	21/15	58	0.79	0.85	0.38	1.84	0.68
**Pain history** (yes/no)											
Last 4 weeks	95	14/81	15	36	33/3	92	**<0.01**	84.36	23.99	424.74	**<0.01**
Last 12 months	95	38/57	40	36	32/4	89	**0.01**	13.01	4.65	46.73	**<0.01**
Ever	95	60/35	63	36	32/4	89	**0.01**	5.25	1.84	19.1	**<0.01**

* Adjusted for age and sex

Table S3: Number of subjects and the predicted probability of their reporting at least one monthly episode of MHC within their first academic year

		**MHC**			**Predicted probabilities**	**Confidence interval**	
						**Lower**	**Upper**
		**No**	**Yes**	**All**			
**Music students**	**Yes**	41	56	97	0.58	0.48	0.68
**Students from other disciplines**	**No**	53	40	93	0.43	0.33	0.54
	**All**	94	96	190	0.51	0.43	0.58

Table S4: univariate longitudinal analysis of risk factors (predictors) for developing MHC

	Monthly episodes: students from other disciplines*					Monthly episodes: music students*					Interaction p-value*
	N	OR	Confidence interval		p-value	N	OR	Confidence interval		p-value	
			Lower	Upper				Lower	Upper		
**Sex**	83	1.73	0.59	5.07	0.32	88	1.05	0.46	2.41	0.91	0.38
**Age**	82	1.05	0.85	1.31	0.63	88	0.95	0.83	1.09	0.5	0.37
**Weight**	83	1.02	0.98	1.07	0.35	87	1	0.96	1.04	1	0.99
**Height**	83	1.01	0.99	1.03	0.8	87	0.99	0.96	1.02	0.75	0.98
**BMI**	83	1.11	0.85	1.44	0.44	87	0.98	0.87	1.11	0.76	0.94
**Sleep duration**	83	0.61	0.29	1.3	0.2	88	0.83	0.49	1.39	0.47	0.42
**Sports**	83	0.97	0.78	1.22	0.82	85	1.01	0.89	1.15	0.84	0.84
**Tobacco use**	83	1.13	0.88	1.47	0.34	51	1.14	0.94	1.4	0.18	0.7
**Practice duration**	NA	NA	NA	NA	NA	82	1	0.97	1.03	0.78	NA
**Experience with primary instrument**	NA	NA	NA	NA	NA	86	0.91	0.82	1.01	0.08	NA
**Health-related quality of life** (SF12)											
Physical functioning	83	0.99	0.88	1.12	0.91	86	0.89	0.84	0.94	**<0.01**	**0.02**
Mental health	83	0.93	0.88	0.99	**0.02**	86	0.99	0.95	1.04	0.71	**0.09**
Pain	82	1.92	0.77	4.78	0.16	88	2.68	1.62	4.42	**<0.01**	0.2
**Anxiety and Depression scale** (HADS)											
Depression	57	1.09	0.94	1.26	0.26	60	1.07	0.88	1.3	0.49	0.57
Anxiety	83	1.17	0.99	1.38	0.06	60	1.07	0.92	1.23	0.37	0.29
**Perfectionism** (FMPS)	NA	NA	NA	NA	NA	33	1.02	1	1.04	0.09	NA
**Stress symptoms** (SCI)	57	1.17	1.06	1.29	**<0.01**	87	1.02	0.94	1.09	0.67	**0.03**
**Performance anxiety** (K-MPAI)	NA	NA	NA	NA	NA	60	0.99	0.98	1.01	0.34	NA

**Hypermobility** (Beighton score)	82	1.13	0.88	1.44	0.35	78	0.97	0.81	1.18	0.77	0.48
**Holding time, front plank** (sec)	82	0.99	0.98	1.01	0.32	75	1	0.99	1.01	0.47	0.72
**Holding time, EEBS test** (sec)	72	1	0.98	1.01	0.47	78	1	0.99	1.01	0.62	0.79
**PPT**	72	0.95	0.9	1	0.05	78	0.99	0.96	1.03	0.7	0.39
**Pain history** (yes/no)											
Last 7 days	73	2.76	0.77	9.9	0.12	88	5.84	2.79	12.19	**<0.01**	0.22
Last 4 weeks	82	1.85	0.61	5.65	0.28	88	3.03	1.39	6.6	**0.01**	0.26
Last 12 months	82	2.92	0.96	8.87	0.06	88	1.98	0.89	4.39	0.09	0.8
Ever	73	2.58	0.71	9.41	0.15	88	2.08	0.89	4.88	0.09	0.95

* Adjusted for age and sex

Statistical analysis (detailed)

The statistical analysis adhered to the analysis strategy described in the pilot study by Ballenberger et al. [23]. All statistical analyses were performed using R (ver. 3.6.3, R Development Core Team, 2020). A p-value of <0.05 was considered significant if not stated otherwise. We compared the study group baseline characteristics and demographic data between two sets of groups (music students vs. students from other disciplines; music students with current MHC with vs. music students without current MHC) using chi-square tests and independent t-tests for categorical and continuous data, respectively. We also determined descriptive statistics such as means, standard deviations, and counts (percentages).

For the baseline analysis, we performed univariate linear regression and logistic regression models for the two comparisons, depending on the data type of the dependent variables (i.e., continuous or dichotomous): First, we compared music students to students from other disciplines with respect to the presence and history of MHC and health-related factors. Second, we compared music students with current MHC to music students without current MHC with respect to health-related factors. Current MHC was defined as described above. We entered MHC-related variables and health-related factors separately as dependent variables and compared the results. The effects are given as standardized mean differences (SMD) and odds ratios (OR), which made it possible to compare effects across different outcome measurements.

The goal of the univariate longitudinal analysis was to identify risk factors (predictors) for the emergence of monthly MHC in music students and compare them to those in students from other disciplines. Here, we employed a general linear mixed model with binomial distribution to take into account the correlated data structure (repeated measurements). Subjects were considered to have random effects. We entered the presence of monthly MHC as the dependent variable, separately entering the history of MHC and health-related factors as independent variables. We then performed the analyses separately for music students and students from other disciplines. The effects are given

as odds ratios. In addition, we modeled the interaction between groups (music students and students from other disciplines) and history of MHC and health-related factors to evaluate whether risk factors for monthly MHC differed significantly between groups. Here, a p-value of <0.1 was considered significant. All univariate regression models were adjusted for the covariates of age and gender, since these were considered potential confounders. The covariates remained in the model if their p-values were <0.1 or if they changed the effect of the independent variable of interest by >10%.

The goal of the multivariate longitudinal analysis was to identify a set of predictors/risk factors for monthly events of MHC in music students. Based on a univariate longitudinal analysis, we entered potential candidate variables for the prediction model if their p-value was <0.1. Then, all candidate variables were entered into a multivariate model (binomial general linear mixed model). In the final model, only variables with p-values <0.05 were retained. The predictive accuracy of the model was determined by leave-one-out validation and calculating the area under the ROC curve (AUCs).

We computed simple cross-tables to compare the monthly prevalence of MHC (number of health complaints experienced in the preceding month) and the number of incident cases (at least one episode of MHC within one year) in music students to students from other disciplines. Predicted probabilities for both monthly prevalence and incident cases of MHC, including confidence intervals, are provided. To predict monthly prevalence, we employed a binomial general linear mixed model, which is capable of taking into account repeated measurements (correlated data). To predict yearly incidence rates, we used a simple logistic regression model. We employed residuals diagnostics and plots to check the validity of all regression models.
